# Long term effect of feeding spray dried plasma during the nursery on subsequent performance and health status to market weight

**DOI:** 10.3389/fvets.2025.1636164

**Published:** 2025-12-15

**Authors:** Caio Abércio da Silva, Kelly Lais de Souza, Cleandro Pazinato Dias, Marco Aurélio Callegari, Joe Crenshaw, Javier Polo, Luis Rangel, Yanbin Shen, Rafael Humberto de Carvalho

**Affiliations:** 1 Department of Animal Science, State University of Londrina, Londrina, Paraná, Brazil; 2 Akei Animal Research, Fartura, São Paulo, Brazil; 3 APC LLC, Ankeny, IA, United States; 4 APC do Brazil, Chapecó, Brazil

**Keywords:** backfat thickness, diarrhea index, growth performance, lean percentage, lunglesions, nursery nutrition, spray-dried plasma, swine

## Abstract

Spray-dried plasma (SDP) is widely used in nursery pig diets due to nutritional and functional benefits, including improved feed intake, gut health, and immune modulation. Although its short-term effects are well documented, its long-term impact on growth and health until market weight remains unclear. We evaluated the effects of increasing SDP inclusion levels during the nursery phase on subsequent performance, health status, and carcass traits in pigs. A total of 300 weaned piglets (PIC® Camborough × AG 415) were randomly allocated to five dietary treatments with cumulative SDP intakes, differing in cumulative SDP intake: control (CON), 0 g SDP; low (L-SDP), 85 g; medium (M-SDP), 180 g; high (H-SDP), 340 g; and very high (VH-SDP), 610 g. Pigs were monitored across nursery, growing, and finishing phases. During the nursery, SDP inclusion had no effect on average daily feed intake (ADFI), average daily gain (ADG), feed conversion rate (FCR), or final body weight (FBW). However, pigs receiving SDP diets showed reduced diarrhea severity (*p* < 0.05) and required fewer medical interventions for gastrointestinal disorders (p < 0.05). In the grow-finish phase, regression analysis revealed a linear increase in ADFI (*p* = 0.074), ADG (*p* = 0.062), and FBW (*p* = 0.037) at slaughter with higher nursery SDP intake. Carcass trait evaluation indicated that increasing nursery SDP intake was associated with an increase in backfat thickness (*p* = 0.052) and a reduction in carcass lean percentage (*p* = 0.039), although lean meat yield (kg) was not different among treatments. Importantly, all SDP-fed groups exhibited a lower lung pneumonia index at slaughter compared with the control (*p* < 0.001). These results support the strategic use of SDP in nursery diets, aiming to promote subsequent health and performance up to commercial slaughter age.

## 1 Introduction

Spray-dried plasma (SDP), derived from bovine, porcine, or mixed sources, is widely incorporated into early-phase diets for weaned piglets, a period characterized by reduced feed intake and compromised intestinal health (1, 2). Common inclusion levels range from 2 to 5%, although specific formulations may reach up to 7 to 8%, depending on production goals and health status of the herd (3, 4).

The beneficial effects of SDP result from both its nutritional composition and physiological properties. Its high palatability enhances feed intake during the post-weaning phase, a period typically marked by reduced consumption (5, 6). This increased intake is supported by the high digestibility of SDP, which is a rich source of lysine (6 to 7%) and other essential amino acids and often surpasses conventional protein supplements in nutritional quality. In addition, SDP supplies essential vitamins and minerals, including calcium, phosphorus, sodium, chlorine, magnesium, and potassium, which are vital for the proper growth and development of piglets (1, 7, 8).

Beyond its nutritional value, SDP contributes to immune modulation and intestinal health in weaned piglets (9). Its composition includes elevated concentrations of immunoglobulins, glycoproteins, growth factors, bioactive peptides, and other biologically active compounds that interact with the gut-associated lymphoid tissue (GALT). These components exert both local and systemic immunomodulatory effects, helping to preserve intestinal integrity and reduce inflammatory responses. By supporting a more stable and functional digestive environment, SDP enhances gut health and immune function, leading to improved well-being and growth performance in piglets (1, 10, 11).

The beneficial effects of SDP on growth performance of pigs post-weaning period and stability against health challenges are well-established (1). These outcomes are strongly influenced by the level of dietary inclusion and the quality of the ingredients used, particularly protein sources. Due to its unique combination of functional and nutritional properties, SDP has been widely recognized as a reference ingredient in studies evaluating dietary strategies for weaned piglets (1, 3).

Although the advantages of SDP in early-phase diets are consistently reflected in improved growth performance and health of nursery pigs, there is limited evidence regarding the persistence of these benefits during later stages of production when SDP no longer included in the diets. Key aspects, such as growth performance, immune response, and carcass traits up to slaughter weight remain underexplored; although positive responses in growth performance have been reported, findings are inconsistent, and only a few studies report favorable effects on intestinal health (12–14). Thus, further investigation is warranted to determine whether the early inclusion of SDP yields sustained improvements throughout the grow-finish period, ultimately contributing to enhanced long-term productivity in swine production systems.

Previous studies investigating the long-term effects of SDP supplementation have primarily focused on growth performance. For instance, Pujols et al. (15) reported that pigs receiving 6% SDP in nursery diets-maintained performance benefits through to slaughter, suggesting prolonged effects of early dietary intervention. Boyer et al. (16) demonstrated enhanced immune responses against *Salmonella typhimurium* with 5% SDP inclusion in weaned-pig diets. Similarly, Campbell et al. (17) observed improved immune responses against swine influenza virus with up to 8% SDP in early post-weaning diets, while Pujols et al. (15) found that 6% SDP in pre-starter diets conferred protective benefits in pigs vaccinated against *PCV2* and *Mycoplasma hyopneumoniae*.

Despite these findings, it remains uncertain whether the health and growth performance benefits conferred by SDP during the nursery phase persist into the grow-finish stages. These effects appear to be dose-dependent, yet the extent to which early benefits scale with inclusion levels and translate into long-term outcomes has not been clearly established. This study tested the hypothesis that increasing cumulative nursery intake of spray-dried plasma through combinations of inclusion level and feeding duration improves growth and health during the nursery phase and that part of this benefit carries over into the grow-finish period, yielding higher average daily gain and final body weight at slaughter, with possible changes in carcass composition. To evaluate this hypothesis, we applied multiple inclusion levels across distinct nursery durations, including late-nursery weeks that are uncommon in commercial practice, and assessed nursery performance and health as well as the persistence of responses through the grow-finish and up to slaughter.

## 2 Materials and methods

### 2.1 Animals, diets, and experimental design

All procedures adopted in this study were previously reviewed and approved by the Animal Research and Experimentation Ethics Committee of Akei Animal Research under protocol number 013.20. A total of 300 weaned pigs (PIC® Camborough × AG 415), including both females and barrows, from three different farms, with an average weaning age of 22 days and an initial body weight of 5.90 ± 0.68 and 5.74 ± 0.81 kg for barrows and females, respectively, were used in the study. Pigs were allocated to pens according to a randomized complete block design, stratified by initial body weight and sex. Five dietary treatment groups were established, each comprising 12 replicate pens (6 replicates per sex), with five pigs of the same sex per pen. The pen served as the experimental unit for growth performance-related variables.

Pens were randomly assigned within each block to one of five nursery feeding programs, consisting of four dietary phases: Pre-starter I (days 0–7), Pre-starter II (days 8–14), Starter I (days 15–21), and Starter II (days 22–42). Each dietary treatment varied in the level of SDP (AP 920®, APC LLC, Araçatuba, São Paulo, Brazil) included across the phases. The treatment groups were defined as follows: the control group (CON) received 0% SDP in all phases. The low-inclusion SDP group (L-SDP) received 3% SDP in Pre-starter I and 2% SDP in Pre-starter II, with no SDP in subsequent phases. The medium-inclusion SDP group (M-SDP) received 5% SDP, 3% SDP, 1% SDP, and 0% SDP across the respective phases; the high-inclusion SDP group (H-SDP) received 7% SDP, 5% SDP, 3% SDP, and 0% SDP; and the very-high-inclusion SDP group (VH-SDP) received 7% SDP, 5% SDP, 3% SDP, and 1.5% SDP.

Diets were provided according to pre-established feed budgets until 64 days of age, as described in Supplementary Table S1. The estimated cumulative intake of SDP per pig during the nursery phase was 0 g (CON), 85 g (L-SDP), 180 g (M-SDP), 340 g (H-SDP), and 610 g (VH-SDP). These values were later used as continuous variables in regression analyses for growth performance and carcass traits. At the end of the nursery period, pigs were transferred to a grow-finish facility, remaining in their original treatment groups and pen compositions, with five pigs per pen. All pigs were fed a common diet throughout the grow-finish phase until reaching slaughter weight. Individual body weights were recorded at the beginning and end of each dietary phase, with the following durations: Pre-starter I (7 days), Pre-starter II (7 days), Starter I (7 days), Starter II (21 days), Growing I (21 days), Growing II (21 days), Finishing I (35 days) and Finishing II (21 days). Measurements were conducted in both the nursery (42 days) and grow-finish (98 days) periods, and feed intake was measured on a pen basis. Pigs had *ad libitum* access to feed and water throughout the experimental period. The ingredient composition and calculated nutrient levels for the Pre-starter I and Pre-starter II diets for each treatment are provided in Supplementary Table S1, and those for the Starter I and Starter II phases are presented in Supplementary Table S2. During the grow-finish period, all pigs received common diets divided into four sequential phases: Grower I, Grower II, Finisher I, and Finisher II. The composition of the grow–finish diets is shown in Supplementary Table S3. All diets within each nursery and grow-finish phase were formulated to be iso-nutrient across treatments.

Health management protocols included a standardized vaccination schedule and preventive treatments. Pigs received two intramuscular doses of a commercial vaccine against *Mycoplasma hyopneumoniae* and porcine circovirus type 2 (Circumvent® PCV M, MSD Animal Health, Boxmeer, the Netherlands; 2 mL per dose), administered at weaning and 21 days later. Additionally, pigs were vaccinated against *Haemophilus parasuis* and *Streptococcus suis* using an autogenous vaccine (TECSA Laboratories, Belo Horizonte, Brazil; 2 mL intramuscular) at 15 and 35 days of age. As part of the preventive antimicrobial program, pigs received four metaphylactic treatments with tiamulin (10 mg/kg BW; Suistin® 50, Vansil Saúde Animal, Descalvado, Brazil) combined with doxycycline (10 mg/kg BW; Farmaxilin® 50, Farmabase Saúde Animal, Jaguariúna, Brazil) during the following age intervals: 29–36, 43–50, 86–93, and 141–148 days. All individual therapeutic treatments administered throughout the wean-to-finish period were recorded.

### 2.2 Data collection

Growth performance was evaluated in each dietary phase using average daily feed intake (ADFI), average daily gain (ADG), and feed conversion rate (FCR), with the pen as the experimental unit. Individual body weight (BW) was recorded weekly and at the start and end of each phase. Feed intake was measured per pen by feed disappearance (feed offered minus refusals) and summed within phases. Performance metrics followed standard definitions: ADG was computed weekly as the change in BW divided by the number of days between weightings, ADFI as pen-level feed disappearance normalized by pig-days, and FCR as the ratio of ADG to ADFI.

During the 42-day nursery phase, fecal consistency was evaluated twice daily according to the methodology described by Yu et al. (18). Fecal scores ranged from 0 to 3, where score 0 indicated firm and normal feces, score 1 represented soft but formed feces, score 2 indicated pasty consistency, and score 3 referred to liquid diarrhea. The diarrhea index was calculated as the ratio of the number of days with score 2 or 3 diarrhea and the total number of observation days for each pen (Diarrhea Index = Days with score 2 or 3/Total study days) (19). Mortality and culling events were recorded throughout the experiment and analyzed by production phase and cumulatively, with probable causes documented when applicable.

### 2.3 Carcass traits

Carcass traits were evaluated following the procedure of Kim et al. (20) and Johnson et al. (21) using a Hennessy Grading Probe 7 (Hennessy Grading Systems, HGP7, New Zealand), with minor adaptations. Measurements were obtained using a Hennessy Grading Probe 7 (Hennessy Grading Systems, HGP7, New Zealand). Measurements of backfat thickness and loin width were obtained 30 min post-slaughter at the anatomical midpoint between the last thoracic vertebra (T14) and the first lumbar vertebra (L1). Carcass yield (%) was calculated as hot carcass weight (HCW) divided by the individually recorded final live BW (kg) on farm and multiplied by 100. Carcass lean content (%) was estimated from HCW (kg), backfat thickness (mm), and loin width (mm) using the manufacturer-specified equation: lean (%) = 54.449–0.5623 × backfat (mm) + 0.198 × loin width (mm), as implemented in the grading probe software (Hennessy Grading Systems, New Zealand). At the end of the performance evaluation, all animals (286 in total, excluding 14 deaths) were sent for slaughter. Of these, 272 carcasses were evaluated, as 14 were partially or totally condemned by the federal inspection service. Each pig was considered one replicate. In addition, lung health was assessed through the Index of Pneumonia (IP), calculated based on macroscopic post-mortem examination of the lungs. The method followed the criteria described by Madec and Kobisch (22), in which each lung lobe was independently evaluated according to its proportional contribution to total lung volume. Lung lobes were scored from 0 to 4 according to the extent of consolidation: 0 = no visible lesions; 1 = 1–25% of the lobe affected; 2 = 26–50%; 3 = 51–75%; and 4 = 76–100%. Scores were used to estimate the overall severity and prevalence of pneumonia in each pig.

### 2.4 Data analysis

#### 2.4.1 Performance data and structure

Performance was recorded at the pen level (five pigs per pen; single-sex pens) with repeated measures across nursery (Pre-starter I, Pre-starter II, Starter I, and Starter II) and grow–finish phases (Grower I, Grower II, Finisher I, and Finisher II). Cumulative nursery, grow–finish, and wean-to-finish outcomes were computed per pen and adjusted for removals using pig-days. Carcass traits were recorded individually at slaughter; pigs remained in their nursery pens throughout rearing, resulting in pen-level clustering.

#### 2.4.2 Linear and mixed models for continuous outcomes

Phase-specific and cumulative performance variables (ADFI, ADG, FCR, and BW) were analyzed using linear mixed-effects models (SAS 9.4; PROC MIXED). For phase-wise analyses, fixed effects were treatment (5 levels), phase, and their interaction (treatment × phase), with sex as an additional fixed factor. Block was included as a random effect, and pen was modeled as a random intercept nested within block, with repeated measures on pen across phases. Sixteen variance–covariance structures (CS, UN, AR (1), ARH (1), TOEP, TOEPH, and heterogeneous variants) were compared, and the best-fitting structure was selected by corrected Akaike’s Information Criterion (AICc), favoring the simpler model when ΔAICc < 2. Degrees of freedom were estimated using the Kenward–Roger method. For cumulative outcomes, analogous models were fitted without the repeated-measures term (random: block and pen; fixed: treatment and sex). When the overall treatment effect was significant (*α* = 0.05), pairwise comparisons used Tukey–Kramer adjustment.

#### 2.4.3 Treatment × sex interaction

Although a significant treatment × sex interaction was initially detected for some performance variables, the interaction lacked biological plausibility and the magnitude of differences between sexes was small relative to within-sex variability. Moreover, excluding the interaction improved model parsimony (ΔAICc < 2). Therefore, results are presented collapsed over sex, with sex retained as a fixed adjustment factor.

#### 2.4.4 Generalized models for binary or proportional outcomes

Mortality, culling, and the proportion of medicated pigs were analyzed at the pen level using binomial generalized linear mixed models (PROC GLIMMIX; logit link). The number of pigs at risk defined the binomial denominator. Treatment and sex were fixed effects; block and pen were random intercepts.

#### 2.4.5 Carcass traits

Carcass variables were analyzed using linear mixed-effects models with treatment and sex as fixed effects, block as a random effect, and pen as a random intercept to account for clustering.

#### 2.4.6 Dose–response analysis

A monotonic dose–response relationship was hypothesized for cumulative nursery SDP intake (g/pig). Following the overall ANOVA, orthogonal polynomial contrasts (linear and quadratic) were tested across the ordered SDP inclusion levels (0, 85, 180, 340, and 610 g/pig). Regression analysis was conducted only when a linear or quadratic contrast indicated a significant (*p* < 0.05) or trending (0.05 ≤ *p* < 0.10) response. Linear models were fitted using pen-level least squares means as dependent variables and cumulative SDP intake as a continuous predictor; quadratic terms were retained only if they improved fit (ΔAICc ≥ 2 and *p* < 0.05). The coefficient of determination (R^2^) was reported for each fitted equation. All analyses were performed in SAS 9.4 (Proc MIXED and Proc GLIMMIX Procedures).

#### 2.4.7 Model diagnostics and assumptions

Residual normality (Shapiro–Wilk, Q–Q plots) and homoscedasticity (studentized residuals vs. fitted values, Levene-type tests) were evaluated for linear models. For GLMMs, Pearson and deviance residuals were examined. Outliers and influential observations were screened via studentized residuals (|*t*| > 3), Tukey’s 1.5 × IQR, and Cook’s D > 4/n; none met exclusion criteria. Leave-one-out checks confirmed model robustness. When assumptions were violated and transformation was unsuitable, the Kruskal–Wallis test followed by Dunn’s multiplicity-adjusted comparisons was applied (e.g., pneumonia index). All analyses were conducted in SAS 9.4 (Proc MIXED and Proc GLIMMIX Procedures). Statistical significance and trends were declared at *p* < 0.05 and 0.05 ≤ *p* < 0.10, respectively.

## 3 Results

In the Pre-starter I phase (Table 1), inclusion of 3% SDP in the L-SDP group was associated with a trend toward a 20.6% increase in ADFI compared to CON (0.158 *vs.* 0.131 kg; *p* = 0.072), although no differences were observed in ADG, FCR, or FBW. Regression analysis for this phase indicated no significant linear or quadratic trends.

**Table 1:** Wean-to-market performance by individual phase, and cumulative nursery, grow–finish, and wean-to-market periods performance of pigs fed diets with variable levels of spray-dried plasma (SDP) per nursery phase.

Performance parameters	Experimental groups					SEM	*p* -value	Regression	
	CON	L-SDP	M-SDP	H-SDP	VH-SDP			Linear	Quadratic
Pre-starter I									
IBW, kg	5.82	5.82	5.81	5.82	5.89	0.097	0.801	NS	NS
ADFI, kg/day	0.131^z^	0.158^y^	0.150^yz^	0.154^yz^	0.155^yz^	0.004	0.072	NS	NS
ADG, kg/day	0.088	0.123	0.100	0.104	0.103	0.005	0.278	NS	NS
FCR	2.198	1.346	1.529	1.560	1.737	0.124	0.198	NS	NS
FBW, kg	6.43	6.68	6.51	6.55	6.61	0.111	0.287	NS	NS
Pre-starter II									
ADFI, kg/day	0.318^b^	0.379^a^	0.364^a^	0.358^a^	0.350^ab^	0.007	0.003	NS	0.005^1^
ADG, kg/day	0.250^b^	0.321^a^	0.322^a^	0.316^a^	0.300^a^	0.007	<0.001	NS	NS
FCR	1.304^z^	1.194^yz^	1.136^y^	1.138^y^	1.169^y^	0.022	0.068	NS	0.024^2^
FBW, kg	8.18^b^	8.93^a^	8.77^a^	8.77^a^	8.71^a^	0.145	<0.001	NS	NS
Starter I									
ADFI, kg/day	0.488	0.512	0.519	0.520	0.499	0.009	0.378	NS	NS
ADG, kg/day	0.386	0.376	0.368	0.367	0.348	0.008	0.210	NS	NS
FCR	1.272^a^	1.364^ab^	1.413^b^	1.422^b^	1.453^b^	0.017	0.009	<0.001^3^	<0.001^4^
FBW, kg	10.89	11.57	11.43	11.34	11.32	0.178	0.112	NS	NS
Starter II									
ADFI, kg/day	0.860	0.905	0.898	0.884	0.929	0.014	0.287	NS	NS
ADG, kg/day	0.550	0.585	0.590	0.566	0.590	0.008	0.207	NS	NS
FCR	1.560	1.544	1.525	1.559	1.568	0.010	0.527	NS	NS
FBW, kg	22.46	23.47	23.75	23.25	23.73	0.328	0.112	NS	NS
Growing I									
ADFI, kg/day	1.547	1.558	1.573	1.562	1.607	0.021	0.798	NS	NS
ADG, kg/day	0.838	0.844	0.858	0.840	0.862	0.009	0.823	NS	NS
FCR	1.843	1.842	1.832	1.852	1.862	0.010	0.800	NS	NS
FBW, kg	40.07	41.17	41.77	40.90	41.85	0.487	0.315	NS	NS
Growing II									
ADFI, kg/day	2.203	2.155	2.240	2.237	2.263	0.026	0.690	NS	NS
ADG, kg/day	1.093	1.057	1.107	1.093	1.086	0.008	0.296	NS	NS
FCR	2.013	2.040	2.020	2.044	2.079	0.016	0.605	NS	NS
FBW, kg	63.03	63.82	65.03	63.88	64.67	0.580	0.315	NS	NS
Finishing I									
ADFI, kg/day	2.737	2.770	2.731	2.88	2.845	0.037	0.587	NS	NS
ADG, kg/day	1.079	1.094	1.065	1.107	1.100	0.008	0.632	NS	NS
FCR	2.531	2.531	2.564	2.598	2.578	0.021	0.824	NS	NS
FBW, kg	100.82	102.11	102.32	102.65	103.18	0.714	0.741	NS	NS
Finishing II									
ADFI, kg/day	2.359	2.428	2.497	2.583	2.585	0.035	0.142	0.025^5^	0.044^6^
ADG, kg/day	0.765	0.806	0.810	0.873	0.863	0.021	0.401	0.079^7^	NS
FCR	3.159	3.060	3.250	2.993	3.084	0.067	0.732	NS	NS
FBW, kg	116.89	118.54	119.43	120.65	120.83	0.787	0.323	0.095^8^	NS
Nursery (wean to end of nursery)									
ADFI, kg/day	0.586	0.627	0.621	0.614	0.632	0.010	0.112	NS	NS
ADG, kg/day	0.396	0.429	0.427	0.415	0.420	0.006	0.113	NS	NS
FCR	1.479	1.460	1.457	1.480	1.499	0.008	0.298	NS	NS
Grow-finishing (end of nursery to market)									
ADFI, kg/day	2.286	2.305	2.328	2.396	2.400	0.024	0.374	0.073^9^	NS
ADG, kg/day	0.961	0.971	0.975	0.996	0.995	0.007	0.345	0.061^10^	NS
FCR	2.375	2.374	2.384	2.403	2.408	0.026	0.912	NS	NS
Total (wean to market cumulative data)									
ADFI, kg/day	1.776	1.802	1.816	1.861	1.870	0.018	0.303	0.068^11^	NS
ADG, kg/day	0.792	0.808	0.811	0.822	0.823	0.005	0.228	0.066^12^	NS
FCR	2.240	2.229	2.238	2.262	2.268	0.013	0.813	NS	NS

Values are least-squares means of 12 pens per treatment. Within rows, means with different superscripts (^a, b^) differ (Tukey–Kramer, *p* < 0.05), and those with (^y, z^) indicate trends (0.05 < *p* ≤ 0.10). CON (0%-SDP): without any SDP; L-SDP (3, 2, 0, 0% SDP in Pre-starter I, Pre-starter II, Starter I, and Starter II): Low SDP; M-SDP (5, 3, 1, 0% SDP in the same phases): Moderate SDP; H-SDP (7, 5, 3, 0%): High SDP; VH-SDP (7, 5, 3, 1.5%): Very High SDP. ADFI = average daily feed intake; ADG = average daily gain; FCR = feed conversion ratio; IBW = initial body weight; FBW = final body weight. Regression equations: ^1^ADFI = 0.2678 + 0.3723·dose − 0.5343·dose^2^ (*R*^2^ = 0.172; *p* = 0.005); ^2^FCR = 1.2845–0.9295·dose − 1.2332·dose^2^ (*R*^2^ = 0.123; *p* = 0.024); ^3^FCR = 1.2902–0.7205·dose − 0.7582·dose^2^ (*R*^2^ = 0.247; *p* < 0.001); ^4^FCR = 1.3246 + 0.2522·dose (*R*^2^ = 0.191; *p* < 0.001); ^5^ADFI (kg) = 2.4013 + 0.3713·dose (*R*^2^ = 0.085; *p* = 0.025); ^6^ADFI (kg) = 2.3554 + 0.9978·dose − 1.0142·dose^2^ (*R*^2^ = 0.105; *p* = 0.044); ^7^ADG (kg) = 0.7808 + 0.1720·dose (*R*^2^ = 0.052; *p* = 0.079); ^8^FW (kg) = 117.7985 + 6.0901·dose (*R*^2^ = 0.048; *p* = 0.095); ^9^ADFI = 0.9660 + 0.0589·dose (*R*^2^ = 0.059; *p* = 0.073); ^10^ADG (kg) = 0.9661 + 0.0589·dose (*R*^2^ = 0.059; *p* = 0.061); ^11^ADFI (kg) = 1.7873 + 0.1577·dose (*R*^2^ = 0.057; *p* = 0.068); ^12^ADG (kg) = 0.8004 + 0.0462·dose (*R*^2^ = 0.057; *p* = 0.066). NS = not significant.

In the Pre-starter II phase, all SDP-fed groups (L-SDP, M-SDP, and H-SDP), except group VH-SDP, had higher ADFI compared with CON (Table 1). L-SDP increased ADFI by 19.2% (0.379 vs. 0.318 kg), M-SDP by 14.5% (0.364 kg), and H-SDP by 12.6% (0.358 kg) (*p* = 0.003) compared with CON. The higher ADFI observed during the Pre-starter II phase was accompanied by higher ADG across all SDP-fed groups relative to CON. ADG increased by 28.4% in L-SDP, 28.8% in M-SDP, 26.4% in H-SDP, and 20.0% in VH-SDP compared to CON (*p* < 0.001). The overall effect on FCR showed a trend (*p* = 0.068) toward improved efficiency, with FCR improvements of 9.1, 7.1, 7.1, and 6.5%, respectively, for the same treatment groups. By the end of this phase, pigs fed L-SDP, M-SDP, H-SDP, and VH-SDP diets had higher FBW than the control group (p < 0.001), showing increases of 9.2, 7.2, 7.2, and 6.5%, respectively.

In the Starter I phase (Table 1), FCR was better in the CON group compared to M-SDP and H-SDP (*p =* 0.009), indicating better feed efficiency in pigs not supplemented with SDP during this phase, but no significant differences were observed between CON and the L-SDP or VH-SDP groups. Regression analysis for the Starter I phase indicated a linear increase in FCR with higher nursery SDP inclusion level (*p =* 0.001).

In the Starter II phase (Table 1), no differences were observed among treatments for ADFI, ADG, or FCR, and no regression effects were detected (*p* > 0.05). Similarly, during the Grower I, Grower II, and Finisher I phases, SDP inclusion during the nursery phase did not influence growth performance outcomes, with no significant differences among treatments for any of the variables evaluated. However, in the Finisher II phase (136 to 166 days of age), a linear trend toward improvement was observed for ADFI (*p* = 0.092), ADG (*p* = 0.079), and final weight (*p* = 0.095), indicating that higher levels of SDP inclusion in the nursery phase were associated with enhanced growth performance in the final stage of production.

Cumulative growth performance results for three periods: nursery, grow–finish, and overall wean-to-market showed that supplementation with SDP during the nursery phase did not produce significant differences among treatments within any cumulative period (Table 1; ANOVA = *p* > 0.05). However, regression analysis indicated linear trends for increased ADFI and ADG with higher cumulative SDP intake during the grow–finish (*p* = 0.073 and 0.061, respectively) and wean-to-market phases (*p* = 0.068 and 0.066, respectively). These findings suggest dose-dependent tendencies rather than confirmed residual treatment effects.

Cumulative growth performance results for three periods: from weaning to the end of the nursery phase, from nursery exit to the end of the grow-finish phase, and the overall wean-to-market period (Table 1), show that, although supplementation with SDP in the nursery phase did not improve growth performance within the nursery itself, positive effects emerged during the grow-finish period and persisted through slaughter. A linear trend was detected for ADFI and ADG during the grow–finish period (*p* = 0.073 and 0.061, respectively) and across the total wean-to-market period (*p* = 0.068 and 0.066, respectively).

Across the nursery phase, the number of cases of diarrhea (scores 2 and 3, and their combined total) was significantly higher in pigs from the CON (0-SDP) and H-SDP groups, whereas the L-SDP and VH-SDP groups had the lowest counts (*p* < 0.001; Table 2). When considering the combined frequency of scores 2 and 3, the incidence of diarrhea in L-SDP, M-SDP, H-SDP, and VH-SDP groups corresponded to 35.6, 51.1, 73.3, and 28.9% of the rate observed in the CON group, respectively.

**Table 2:** Effects of diets with different levels of spray-dried plasma (SDP) on diarrhea occurrence and score, and diarrhea index in piglets during the nursery phase (the values are expressed in number of piglets with diarrhea).

Diarrhea score	Experimental groups					
	CON	L-SDP	M-SDP	H-SDP	VH-SDP	*p*-value
Piglets with diarrhea score 2, *n*	7^b^	1^a^	5^b^	6^b^	3^b^	<0.001
Piglets with diarrhea score 3, *n*	19^b^	7^a^	10^a^	14^ab^	12^a^	<0.001
Total piglets with diarrhea (scores 2 + 3), *n*	26^b^	8^a^	15^ab^	20^b^	13^a^	<0.001
Index	0.45	0.16	0.23	0.33	0.21	–

Values for scores 2 and 3 (and Total) are counts of pigs with at least one episode during the 42-day nursery period. Within rows, values with different superscripts (^a, b^) differ (*p* < 0.05) based on a *binomial GLMM (logit link). CON (0%-SDP): without any SDP; L-SDP (3, 2, 0, 0% SDP in Pre-starter I, Pre-starter II, Starter I, and Starter II): Low SDP; M-SDP (5, 3, 1, 0% SDP in the same phases): Moderate SDP; H-SDP (7, 5, 3, 0%): High SDP; VH-SDP (7, 5, 3, 1.5%): Very High SDP.

Throughout the experimental period, the pigs were affected by various diseases and received individualized treatments. For diarrhea, pigs in the CON group required a greater number of treatments (*p* < 0.001) compared to pigs fed diets with SDP, with diarrhea being the most common cause of treatment (Table 3). At slaughter, a total of 272 pigs were evaluated for carcass traits, a number lower than the initial 300 pigs assessed for growth performance. This reduction was due to mortality, removals during the study, or loss of identification and traceability during slaughter processing. Despite these losses, mortality and culling rates (Table 4) did not differ among treatments (*p* > 0.05), indicating that the inclusion levels of SDP in nursery diets did not affect overall survivability or removal rates throughout the production cycle.

**Table 3:** Number of pigs affected by diseases and treated with medication according to dietary spray-dried plasma (SDP) levels.

Diseases	Experimental groups					*p*-value
	CON	L-SDP	M-SDP	H-SDP	VH-SDP	
Arthritis, *n*	1	1	5	5	0	0.605
Diarrhea, *n*	33^b^	9^a^	18^a^	23^a^	11^a^	<0.001
Ileitis, *n*	3	0	0	0	0	0.708
Pneumonia, *n*	10	7	10	11	13	0.897
Prolapse, *n*	1	0	0	0	0	0.980

Values are counts of pigs affected during the wean-to-finish period. Within rows, values with different superscripts (^a, b^) differ (*p* < 0.05). *Analyses used binomial GLMMs (logit link). CON (0%-SDP): without any SDP; L-SDP (3, 2, 0, 0% SDP in Pre-starter I, Pre-starter II, Starter I, and Starter II): Low SDP; M-SDP (5, 3, 1, 0% SDP in the same phases): Moderate SDP; H-SDP (7, 5, 3, 0%): High SDP; VH-SDP (7, 5, 3, 1.5%): Very High SDP.

**Table 4:** Effects of dietary spray-dried plasma (SDP) inclusion levels on the number of pigs culled or deceased throughout the experimental period.

Parameter	Experimental groups					*p*-value*
	CON	L-SDP	M-SDP	H-SDP	VH-SDP	
Culled, *n*	2	0	1	0	6	0.869
Dead, *n*	2	0	0	1	2	0.874

Values are counts of pigs removed during the wean-to-finish period. *Analyses used binomial GLMMs (logit link). CON (0%-SDP): without any SDP; L-SDP (3, 2, 0, 0% SDP in Pre-starter I, Pre-starter II, Starter I, and Starter II): Low SDP; M-SDP (5, 3, 1, 0% SDP in the same phases): Moderate SDP; H-SDP (7, 5, 3, 0%): High SDP; VH-SDP (7, 5, 3, 1.5%): Very High SDP.

Increasing SDP inclusion levels during the nursery phase had a positive linear effect on final body weight at slaughter (*p* = 0.037). This improvement in slaughter weight was associated with a trend toward a linear increase in backfat thickness (*p* = 0.052) and a corresponding linear decrease in carcass lean percentage (*p* = 0.039) (Table 5).

**Table 5:** Carcass traits of pigs fed diets with different levels of spray-dried plasma (SDP).

Parameters	Experimental groups					SEM	*p*-value*	Regression	
	CON	L-SDP	M-SDP	H-SDP	VH-SDP			Linear	Quadratic
FW, kg	117.68	118.53	120.17	121.03	121.37	0.560	0.118	0.024^1^	0.045^2^
CW, kg	84.39	84.65	85.79	85.56	85.93	0.452	0.679	NS	NS
CY, %	71.84	71.40	71.38	70.67	70.80	0.188	0.354	NS	NS
BF, mm	15.900	15.918	16.063	17.284	17.327	0.262	0.121	0.024^3^	0.073^4^
LD, mm	57.850	57.857	58.342	58.908	57.034	0.598	0.849	NS	NS
PLM, %	56.987	56.969	56.984	56.411	56.015	0.217	0.623	0.039^5^	NS
KLM, Kg	48.099	48.191	48.825	48.296	48.100	0.302	0.904	NS	NS

Values are least-squares means at the individual carcass level. *Analyses used linear mixed models. CON (0%-SDP): without any SDP; L-SDP (3, 2, 0, 0% SDP in Pre-starter I, Pre-starter II, Starter I, and Starter II): Low SDP; M-SDP (5, 3, 1, 0% SDP in the same phases): Moderate SDP; H-SDP (7, 5, 3, 0%): High SDP; VH-SDP (7, 5, 3, 1.5%): Very High SDP. FW = final weight; CW = carcass weight; CY = carcass yield; BF = backfat; LD = loin depth; PLM = percentage of lean meat; KLM = kilograms of lean meat. The number of carcasses evaluated were: CON = 49; L-SDP = 54; M-SDP = 54; H-SDP = 52; VH-SDP = 46. Regression equations: ^1^FW = 118.3013 + 6.1154·dose (*R*^2^ = 0.015; *p* = 0.024); ^2^FW = 117.5782 + 15.7109·dose − 15.5954·dose^2^ (*R*^2^ = 0.015; *p* = 0.024); ^3^BF = 15.8108 + 2.5825·dose (*R*^2^ = 0.015; *p* = 0.024); ^4^BF = 15.6750 + 4.6549·dose − 2.9294·dose^2^ (*R*^2^ = 0.019; *p* = 0.073); ^5^PLM = 55.1364–2.2502·dose (*R*^2^ = 0.015; *p* = 0.039). NS, not significant.

As the dietary inclusion of SDP increased, the pneumonia index, indicative of lung lesions, decreased (*p* < 0.001). Mean pneumonia index values were 2.201 for CON, 0.875 for L-SDP, 0.635 for M-SDP, 0.576 for H-SDP, and 0.757 for VH-SDP, with all SDP groups significantly lower than CON. The CON group exhibited the highest score; reductions relative CON were 60.2% (L-SDP), 71.2% (M-SDP), 73.8% (H-SDP), and 65.6% (VH-SDP).

## 4 Discussion

The inclusion of SDP in nursery pig diets is well-established for its positive effects on feed palatability, immune modulation, and the functional benefits conferred by glycoproteins and immunoglobulins (23, 24). These mechanisms contribute to improved weight gain, largely due to the high digestibility and nutritional quality of SDP, as well as its beneficial effects on intestinal integrity and function (1). An important factor underlying this response is the modulation of the gut microbiota, as demonstrated by Tran et al. (25), in which pigs fed diets with SDP showed an increase in lactic acid-producing bacteria (*Lactobacillus delbrueckii*) and cellulolytic bacteria (*Ruminococcus albus; Clostridium thermocellum; Clostridium saccharoperbutylacetonicum; Clostridium beijerinckii*; and *Megasphaera elsdenii*), as well as a reduction in the abundance of *Clostridium difficile* compared to control pigs (fed diets without SDP). The authors attributed this modulation to improved dietary protein utilization efficiency and an improved FCR, ultimately leading to greater weight gain during the nursery phase (26–28). The present study corroborates these findings, particularly in the early post-weaning period, confirming that SDP promotes enhanced nutrient absorption and overall growth performance in weaned piglets.

The growth performance improvements observed during the nursery phase, especially in the Pre-starter II phase, are consistent with established benefits of SDP and with recommended inclusion levels reported in previous research (15, 29). Pujols et al. (15) reported that the use of 6% SDP in pre-starter diets during the first 14 days post-weaning enhanced growth performance, a strategy aligned with our findings. Meta-analyses conducted by Balan et al. (1) and Remus et al. (3) also reported increases of approximately 20% in ADFI and 10% in ADG in pigs fed SDP, values comparable to those observed in the L-SDP and M-SDP groups of our study. These results remain consistent even when SDP is included in more complex diets, such as those used in the present study, as previously described by Collins et al. (30) and Wang et al. (31). The functional benefits of SDP are typically observed at inclusion levels between 2 and 8% (3, 4), although some recent protocols have adopted higher rates of 9–10% (1, 32). Importantly, the magnitude of the response to SDP is influenced by the type and quality of the protein sources used in control diets. Diets formulated with lower-quality proteins tend to highlight the beneficial effects of SDP, while high-quality protein sources may attenuate these responses (1). This likely explains the lack of additional growth performance gains observed with higher SDP doses in the Pre-starter I and II phases of our study, where the control diets were nutritionally complex. These findings confirm the effectiveness of SDP during the nursery period, while its influence beyond this phase remains to be interpreted cautiously based on subsequent performance trends.

Our findings are consistent with those reported by Balan et al. (1), Remus et al. (3), and Ruckman et al. (33), who observed that increasing SDP levels beyond a certain threshold did not yield proportional improvements in growth performance. These results support the notion that inclusion levels between 3 and 7% are effective in optimizing growth performance, while higher levels may not confer additional benefits.

Our results challenge the hypothesis of a strictly dose-dependent effect of SDP on growth performance, as previously suggested by Balan et al. (1). This divergence from expected outcomes may be attributed to the high nutritional quality and complexity of the pre-starter control diets (CON, 0-SDP) used in the present study. In this context, our findings are consistent with those reported by Castelo et al. (32), who evaluated nursery diets with 0.0, 3.0, 6.0%, or 9.0% SDP from weaning to 35 days of age, followed by 0.0, 1.5, 3.0%, or 4.5% SDP from 36 to 49 days, and a common SDP-free diet from 50 to 59 days. Similar to our observations, their study did not demonstrate a proportional improvement in growth performance with increasing SDP levels, reinforcing the concept that higher dosages may not consistently yield superior outcomes, particularly when baseline diets are already nutritionally well-balanced.

During the Initial Phase I, only one difference (*p* = 0.009) was observed between treatments for FCR, with the plasma-free treatment (CON) being better than the M-SDP, H-SDP, and SH-SDP treatments, but similar to the L-SDP group (Table 1). Additionally, a linear increase in FCR was detected in the Initial Phase I, which may reflect a compensatory effect after the improvement in growth and feed efficiency observed in the preceding Pre-Initial Phase I. This pattern aligns with the compensatory growth phenomenon described by Skiba (34), in which pigs previously experiencing superior nutrient utilization or feed restriction exhibit transient reductions in efficiency in subsequent phases. Despite the fact that all experimental diets had the same nutritional and energy composition, the feed restriction observed in the CON group in the first 2 weeks after weaning may be characterized as a qualitative restriction (35, 36). In our study, the early advantage provided by SDP in the initial nursery phase may have led to a temporary compensatory response in the CON group, ultimately affecting feed efficiency dynamics during Starter I.

The absence of significant differences between the control group (CON) and the treatments including SDP (L-SDP, M-SDP, H-SDP and SH-SDP) during the initial phase II suggests that the physiological and immunological immaturity of the gastrointestinal tract (37), which is most critical during the first 15 days post-weaning (38, 39), had already improved by this stage. As a result, the impact of SDP inclusion became less pronounced, supporting evidence that its greatest benefits are concentrated during the first and second weeks after weaning, with minimal or no effects in subsequent weeks (1). This finding reinforces the role of SDP in facilitating early post-weaning adaptation by improving feed intake, intestinal health, and immune responsiveness during the critical transition from milk to solid feed. Once digestive and immune functions are more fully developed, the benefits of SDP become less relevant, highlighting the importance of its strategic use in early-phase diets to maximize growth performance outcomes (1).

Additionally, the naturally improved immune status of older pigs in the Starter I and II phases contributes to greater stability against health challenges, reducing the reliance on functional ingredients such as SDP (17, 40, 41). The efficacy of SDP is known to be greater under low sanitary and high environmental stress, where its immunomodulatory and gut-supportive properties enhance growth performance (4, 42). As pigs mature, the development of their adaptive immune system enables them to better manage physiological stressors without dietary intervention. These observations support the concept that SDP inclusion is most beneficial during early post-weaning, particularly when piglets are more vulnerable to intestinal disturbances, immune suppression, and environmental fluctuations (38). Evaluating feed intake, particularly in the first two nursery phases, suggests that the superior intake with SDP diets may explain subsequent improvements in growth and performance during the grow–finish period. This hypothesis is based on some studies (43, 44) which piglets with high intakes of highly digestible feeds during the 14 days after weaning show concomitant increases in weight gain during this phase (43–45), and these gains persist into later nursery phases.

The cost-effectiveness of different SDP inclusion levels must be carefully evaluated, especially in pre-starter diets, which are typically the most expensive component of nursery feeding programs. In this context, the inclusion rates adopted in the present study are consistent with recommendations from previous research (3, 33), which advocate optimized levels that balance growth performance gains with economic feasibility (46). Considering that pre-starter diets account for a substantial portion of total feeding costs, the use of SDP should be strategically timed to coincide with the period of post-weaning vulnerability (37).

During the growing and finishing phases, as well as across the overall experimental period (Table 1), trend-level linear responses were detected for ADFI and ADG, suggesting that early inclusion of SDP may be associated with improved physiological readiness entering the grow–finish phase. However, these trends did not reach statistical significance at the cumulative level. This observation is consistent with the concept that establishing a solid nutritional and immune foundation early in life can favor subsequent performance potential, as suggested by Messier et al. (47) and Pujols et al. (15). The early benefits of SDP—particularly those related to gut health, nutrient absorption, and immune modulation (23–25)—may therefore have supported transient improvements that align with known physiological mechanisms, though not confirmed statistically in later stages. Collectively, these results reinforce the strategic value of early-life nutritional interventions for enhancing resilience and stability during the production cycle (1, 9, 48, 49).

Additionally, the high nutritional value of SDP and its role in promoting gut development contribute to improved gastrointestinal morphology and function (1, 5, 50). Combined with the early establishment of a stable and diverse gut microbiota, these effects support faster physiological maturation of the gastrointestinal tract, which favors efficient nutrient absorption and utilization. Such improvements in digestive capacity during the nursery phase may help interpret the trend-level responses observed later in growth performance, being consistent with previous hypotheses that early-life nutritional strategies can influence subsequent metabolic development (51–53). However, in the present study, these effects were not statistically significant in cumulative analyses, indicating that the influence of SDP was primarily confined to the nursery period.

The inclusion of SDP in piglet diets has been associated with reduced immune system activation, allowing dietary amino acids that would otherwise be directed toward immune cell synthesis to be utilized for growth and tissue development instead (54). This state of immune homeostasis in the intestinal mucosa contributes to the downregulation of pro-inflammatory cytokines, thereby supporting gut health and enhancing overall growth performance (9, 11, 50). These benefits of SDP supplementation were also evident as a reduction in diarrhea incidence throughout the nursery period. This outcome likely reflects the immunoprotective and functional properties of SDP, which contains high levels of immunoglobulins, glycoproteins, growth factors, bioactive peptides, and other biologically active components. These molecules interact with gut-associated lymphoid tissue (GALT), modulating the intestinal immune response and reducing inflammation. By maintaining gut integrity and promoting immune protection, SDP contributes to a more stable digestive environment, reducing the incidence of gastrointestinal disorders and enhancing overall health and growth performance (1, 11).

Previous studies have indicated that SDP may contribute to gut health by supporting intestinal integrity and promoting a balanced microbiota, which is associated with improved gut function and health (5, 50, 55). Although these mechanisms were not directly assessed in the present study, polynomial contrast analysis and subsequent regression indicated only trend-level, positive linear associations between early SDP supplementation and ADFI and ADG during the grow–finish and overall wean-to-market periods. These relationships should therefore be interpreted as non-significant trends rather than evidence of residual treatment effects beyond the nursery phase.

Pigs that received SDP during the nursery phase exhibited a numerical, trend-level increase in body weight at the end of the finishing phase (Table 1). Similarly, slaughter weight and backfat thickness tended to increase with higher nursery SDP inclusion, accompanied by a proportional numerical reduction in carcass lean percentage. This pattern is consistent with the well-documented relationship between heavier pigs and greater fat deposition coupled with lower lean meat yield (56, 57). Despite these numerical shifts, total kilograms of lean meat were unaffected, indicating that higher final weights were not associated with changes in muscle accretion. These tendencies are directionally similar to those reported by Pujols et al. (15), who observed heavier carcasses and slightly lower lean percentages in pigs fed 6% SDP during the first 14 days post-weaning, without differences in total lean mass.

The lower pneumonia index observed in pigs fed diets containing SDP compared with the control group suggests a potential immunomodulatory benefit of this ingredient. *Mycoplasma hyopneumoniae*, the primary pathogen associated with enzootic pneumonia, induces chronic pulmonary lesions characterized by cranioventral lung consolidation (58), a pattern also noted in the present study. Such lesions are known to impair growth performance by reducing feed efficiency and increasing the risk of secondary infections (59). The observed reduction (*p* < 0.001) in pneumonia prevalence among SDP-fed pigs is consistent with the proposed immune-supportive properties of SDP, although this response should be interpreted as indicative rather than conclusive evidence of improved respiratory health.

Early colonization of the respiratory tract by *Mycoplasma hyopneumoniae* is commonly attributed to vertical transmission from the sow during lactation (60). Given this early exposure, the numerically lower incidence of pulmonary lesions in pigs fed SDP during the nursery phase may reflect local and systemic immunomodulatory effects of SDP. These responses are thought to involve stimulation of GALT, which interacts with peripheral immune sites, including the respiratory tract (10, 11). By enhancing mucosal immunity, SDP could transiently increase resistance to respiratory pathogens, thereby complementing its well-established role in maintaining intestinal health. The mechanisms underlying these effects remain incompletely defined. Plasma immunoglobulins can resist gastric digestion and reach the intestinal lumen, where they bind antigens and help prevent intestinal damage (4). Other studies suggest that multiple SDP components may support efficient immune responses to pathogens or stressors, with effects reported in the gastrointestinal (61), reproductive (62), and respiratory systems (11, 40, 41). Collectively, these observations indicate that early-life nutritional interventions such as SDP can transiently enhance immune competence and disease resilience during the nursery period, though in the present study these effects were not statistically sustained into later production stages.

The reduction in pulmonary lesions observed in the present study is directionally consistent with previous research comparing pigs fed SDP to those vaccinated against PCV2 and *Mycoplasma hyopneumoniae*. Pujols et al. (15) reported lower mortality rates in pigs that received SDP for the first 15 days post-weaning, with beneficial effects extending through the production period up to 145 days of age. Although such persistence was not statistically confirmed in the present study, both investigations support the hypothesis that early SDP feeding may reduce the severity of respiratory challenges. Similarly, Campbell et al. (15), demonstrated that pigs experimentally infected with swine influenza virus (SIV) and fed 8% SDP for 28 days post-weaning exhibited fewer lung lesions at the end of the treatment period, reinforcing the potential of SDP to transiently mitigate respiratory inflammation under infectious pressure.

In summary, including SDP in nursery diets reduced diarrhea incidence during the nursery phase and was associated with a lower pneumonia prevalence at slaughter. The most pronounced improvements occurred early after weaning, whereas later responses were limited to non-significant, trend-level associations between nursery SDP intake and final body weight, consistent with the low *R*^2^ values observed in the dose–response regressions. Increasing SDP inclusion also showed a dose–dependent association with greater fat deposition and a lower carcass lean percentage, while total lean meat yield remained unaffected. Overall, these results indicate that SDP supplementation during the nursery phase enhances early post-weaning performance and health, and may contribute to improved overall production efficiency when strategically applied in swine feeding programs.

## Data Availability

The raw data supporting the conclusions of this article will be made available by the authors, without undue reservation.
