# Supplementary material for: Long term effect of feeding spray dried plasma during the nursery on subsequent performance and health status to market weight
Source: Front Vet Sci. 2025 Dec 15;12:1636164. doi: 10.3389/fvets.2025.1636164 (PMC12745488; doi:10.3389/fvets.2025.1636164)
Supplement: Supplementary file 1 [file Table_1.docx]

**Supplementary Table S1**. Ingredient and nutrient composition (as-fed matter basis) of Pre-starter I and II diets by treatment groups.

|  | | | Pre-starter I | | | | | Pre-starter II | | | | |  |
| --- | --- | --- | --- | --- | --- | --- | --- | --- | --- | --- | --- | --- | --- |
| **Ingredients, g/kg** | CON | L-SDP | | M-SDP | H-SDP | VH-SDP | CON | | L-SDP | M-SDP | H-SDP | VH-SDP | |
| Corn, 7.88% CP | 394.4 | 408.3 | | 428.1 | 447.9 | 447.9 | 465.2 | | 473.8 | 488.5 | 497.2 | 497.2 | |
| Soy protein concentrate | 50.0 | 50.0 | | 50.0 | 50.0 | 25.0 | 25.0 | | 25.0 | 25.0 | 25.0 | 25.0 | |
| Soybean meal, 45% CP | 200.0 | 166.6 | | 133.3 | 100.0 | 100.0 | 225.0 | | 203.4 | 181.7 | 160.0 | 160.0 | |
| Yeast extract | 25.0 | 25.0 | | 25.0 | 25.0 | 25.0 | 25.0 | | 25.0 | 25.0 | 25.0 | 25.0 | |
| Spray-dried plasma (SDP)^1^ | 0.0 | 30.0 | | 50.0 | 70.0 | 70.0 | 0.0 | | 20.0 | 30.0 | 50.0 | 50.0 | |
| Milk whey | 214.3 | 214.3 | | 214.3 | 214.3 | 214.3 | 142.9 | | 142.9 | 142.9 | 142.9 | 142.9 | |
| Soybean oil | 46.7 | 41.5 | | 37.4 | 33.3 | 33.3 | 44.7 | | 41.3 | 38.9 | 35.5 | 35.5 | |
| Sugar | 25.0 | 25.0 | | 25.0 | 25.0 | 25.0 | 25.0 | | 25.0 | 25.0 | 25.0 | 25.0 | |
| Vitamin premix^2^ | 2.0 | 2.0 | | 2.0 | 2.0 | 2.0 | 2.0 | | 2.0 | 2.0 | 2.0 | 2.0 | |
| Mineral premix^3^ | 1.0 | 1.0 | | 1.0 | 1.0 | 1.0 | 1.0 | | 1.0 | 1.0 | 1.0 | 1.0 | |
| Phytase (10,000 FTU)^4^ | 0.05 | 0.05 | | 0.05 | 0.05 | 0.05 | 0.05 | | 0.05 | 0.05 | 0.05 | 0.05 | |
| Antioxidant | 6.00 | 6.00 | | 6.00 | 6.00 | 6.00 | 6.00 | | 6.00 | 6.00 | 6.00 | 6.00 | |
| Dicalcium phosphate | 8.60 | 6.90 | | 5.80 | 4.73 | 4.73 | 8.39 | | 7.21 | 6.72 | 5.54 | 5.54 | |
| Limestone | 2.20 | 3.40 | | 4.21 | 5.02 | 5.02 | 3.66 | | 4.48 | 4.89 | 5.70 | 5.70 | |
| Salt | 4.50 | 2.90 | | 1.75 | 0.65 | 0.65 | 4.49 | | 3.39 | 2.84 | 1.73 | 1.73 | |
| Copper sulfate | 0.40 | 0.40 | | 0.40 | 0.40 | 0.40 | 0.40 | | 0.40 | 0.40 | 0.40 | 0.40 | |
| Zinc oxide | 3.50 | 3.50 | | 3.50 | 3.50 | 3.50 | 3.50 | | 3.50 | 3.50 | 3.50 | 3.50 | |
| L-Lysine – HCl 80% | 5.70 | 4.60 | | 4.16 | 3.73 | 3.73 | 6.60 | | 5.81 | 5.75 | 4.97 | 4.97 | |
| DL-Methionine 98% | 3.10 | 2.70 | | 2.50 | 2.33 | 2.33 | 3.31 | | 3.03 | 2.99 | 2.72 | 2.72 | |
| L-Threonine 98% | 3.30 | 2.60 | | 2.34 | 2.05 | 2.05 | 3.56 | | 3.11 | 3.03 | 2.58 | 2.58 | |
| L-Tryptophan 98% | 0.85 | 0.70 | | 0.70 | 0.68 | 0.68 | 0.93 | | 0.85 | 0.86 | 0.78 | 0.78 | |
| L-Valine 98% | 2.10 | 1.40 | | 1.12 | 0.84 | 0.84 | 2.32 | | 1.85 | 1.79 | 1.32 | 1.32 | |
| L-Isoleucine 99% | 0.72 | 0.70 | | 0.88 | 1.06 | 1.06 | 0.98 | | 0.96 | 1.13 | 1.11 | 1.11 | |
| Biocholine 60% | 0.25 | 0.25 | | 0.25 | 0.25 | 0.25 | 0.25 | | 0.25 | 0.25 | 0.25 | 0.25 | |
| Palatability Enhancer | 0.30 | 0.30 | | 0.30 | 0.30 | 0.30 | 0.30 | | 0.30 | 0.30 | 0.30 | 0.30 | |
| Nutrients |  |  | |  |  |  |  | |  |  |  |  | |
| Metabolizable energy, kcal/kg | 3500 | 3500 | | 3500 | 3500 | 3500 | 3475 | | 3475 | 3475 | 3475 | 3475 | |
| Crude fat, % | 7.00 | 6.53 | | 6.18 | 5.83 | 5.83 | 6.91 | | 6.60 | 6.41 | 6.10 | 6.10 | |
| Crude fiber, % | 1.88 | 1.74 | | 1.60 | 1.47 | 1.47 | 2.07 | | 1.98 | 1.89 | 1.80 | 1.80 | |
| Lactose, % | 15.0 | 15.0 | | 15.0 | 15.0 | 15.0 | 10.0 | | 10.0 | 10.0 | 10.0 | 10.0 | |
| Crude protein, % | 19.8 | 20.5 | | 20.7 | 20.8 | 20.8 | 19.3 | | 19.8 | 19.7 | 20.2 | 20.2 | |
| Total lysine, % | 1.54 | 1.56 | | 1.57 | 1.59 | 1.59 | 1.54 | | 1.55 | 1.56 | 1.58 | 1.58 | |
| Digestible Lysine, %^4^ | 1.43 | 1.43 | | 1.43 | 1.43 | 1.43 | 1.43 | | 1.43 | 1.43 | 1.43 | 1.43 | |
| Digestible Methionine, % | 0.55 | 0.51 | | 0.48 | 0.46 | 0.46 | 0.57 | | 0.54 | 0.53 | 0.50 | 0.50 | |
| Digestible Met + Cys, % | 0.86 | 0.86 | | 0.86 | 0.86 | 0.86 | 0.86 | | 0.86 | 0.86 | 0.86 | 0.86 | |
| Digestible Threonine, % | 0.95 | 0.95 | | 0.95 | 0.95 | 0.95 | 0.95 | | 0.95 | 0.95 | 0.95 | 0.95 | |
| Digestible Tryptophan, % | 0.29 | 0.29 | | 0.29 | 0.29 | 0.29 | 0.29 | | 0.29 | 0.29 | 0.29 | 0.29 | |
| Digestible Arginine, % | 1.15 | 1.16 | | 1.14 | 1.11 | 1.11 | 1.10 | | 1.11 | 1.09 | 1.10 | 1.10 | |
| Digestible Valine, % | 0.98 | 0.98 | | 0.98 | 0.98 | 0.98 | 0.98 | | 0.98 | 0.98 | 0.98 | 0.98 | |
| Digestible Isoleucine, % | 0.78 | 0.78 | | 0.78 | 0.78 | 0.78 | 0.78 | | 0.78 | 0.78 | 0.78 | 0.78 | |
| Digestible Leucine, % | 1.39 | 1.49 | | 1.53 | 1.57 | 1.57 | 1.37 | | 1.44 | 1.45 | 1.52 | 1.52 | |
| Digestible Histidine, % | 0.45 | 0.48 | | 0.50 | 0.52 | 0.52 | 0.44 | | 0.46 | 0.47 | 0.49 | 0.49 | |
| Digestible Phenylalanine, % | 0.75 | 0.80 | | 0.81 | 0.82 | 0.82 | 0.75 | | 0.78 | 0.78 | 0.81 | 0.81 | |
| Total Calcium, % | 0.75 | 0.75 | | 0.75 | 0.75 | 0.75 | 0.75 | | 0.75 | 0.75 | 0.75 | 0.75 | |
| Total Phosphorus, % | 0.73 | 0.73 | | 0.73 | 0.73 | 0.73 | 0.69 | | 0.69 | 0.69 | 0.69 | 0.69 | |
| Standardized available phosphorus, % | 0.55 | 0.55 | | 0.55 | 0.55 | 0.55 | 0.50 | | 0.50 | 0.50 | 0.50 | 0.50 | |
| Sodium, % | 0.35 | 0.35 | | 0.35 | 0.35 | 0.35 | 0.30 | | 0.30 | 0.30 | 0.30 | 0.30 | |
| Copper, mg/kg | 105 | 105 | | 105 | 104 | 104 | 106 | | 105 | 105 | 105 | 105 | |
| Zinc, mg/kg | 2574 | 2573 | | 2572 | 2571 | 2571 | 2211 | | 2211 | 2210 | 2209 | 2209 | |
| Choline, mg/kg | 378 | 402 | | 419 | 435 | 435 | 378 | | 394 | 402 | 419 | 419 | |

^1^ SDP, spray-dried plasma (AP920, APC do Brasil). ^2^Vitamin premix provided per kg of diet: 6,000 IU vitamin A; 1,500 IU vitamin D3; 15 mg vitamin E; 1.5 mg vitamin K3; 1.35 mg vitamin B1; 4 mg vitamin B2; 2 mg vitamin B6; 20 μg vitamin B12; 20 mg; 9.35 mg pantothenic acid; 600 μg folic acid; 80 μg biotin; 300 μg Se. ^3^Mineral premix provided per kg of diet: 100 mg Fe; 10 mg Cu; 40 g Mn; 1 mg Co; 100 mg Zn; 1.5 mg I. ^3^Product activity.  ^5^Digestible amino acids values are expressed as standardized ileal digestible. CON = control group; L-SDP = low-inclusion spray-dried plasma; M-SDP = medium-inclusion spray-dried plasma; H-SDP = high-inclusion spray-dried plasma; VH-SDP = very-high-inclusion spray-dried plasma.

**Supplementary Table S2**. Ingredient and nutrient composition (as-fed matter basis) of Starter I and II diets by treatment groups.

|  | Starter I | | | | | Starter II | | | | | |
| --- | --- | --- | --- | --- | --- | --- | --- | --- | --- | --- | --- |
| **Ingredients, g/kg** | CON | L-SDP | M-SDP | H-SDP | VH-SDP | | CON | L-SDP | M-SDP | H-SDP | VH-SDP |
| Corn, 7.88% CP | 509.5 | 509.5 | 522.6 | 529.7 | 529.7 | | 578.9 | 578.9 | 578.9 | 578.9 | 589.0 |
| Soybean meal 45% CP | 280.0 | 280.0 | 260.0 | 240.0 | 240.0 | | 320.0 | 320.0 | 320.0 | 320.0 | 300.0 |
| Yeast extract | 25.0 | 25.0 | 25.0 | 25.0 | 25.0 | | 0.0 | 0.0 | 0.0 | 0.0 | 0.0 |
| Spray-dried plasma (SDP)^1^ | 0.0 | 0.0 | 10.0 | 30.0 | 30.0 | | 0.0 | 0.0 | 0.0 | 0.0 | 15.0 |
| Milk whey | 71.4 | 71.4 | 71.4 | 71.4 | 71.4 | | 0.0 | 0.0 | 0.0 | 0.0 | 0.0 |
| Soybean oil | 44.3 | 44.3 | 42.1 | 38.7 | 38.7 | | 36.9 | 36.9 | 36.9 | 36.9 | 34.1 |
| Sugar | 25.0 | 25.0 | 25.0 | 25.0 | 25.0 | | 25.0 | 25.0 | 25.0 | 25.0 | 25.0 |
| Vitamin premix^2^ | 1.5 | 1.5 | 1.5 | 1.5 | 1.5 | | 1.5 | 1.5 | 1.5 | 1.5 | 1.5 |
| Mineral premix^3^ | 1.0 | 1.0 | 1.0 | 1.0 | 1.0 | | 1.0 | 1.0 | 1.0 | 1.0 | 1.0 |
| Phytase (10,000 FTU)^4^ | 0.05 | 0.05 | 0.05 | 0.05 | 0.05 | | 0.05 | 0.05 | 0.05 | 0.05 | 0.05 |
| Antioxidant | 5.00 | 5.00 | 5.00 | 5.00 | 5.00 | | 0.15 | 0.15 | 0.15 | 0.15 | 0.15 |
| Dicalcium phosphate | 7.99 | 7.99 | 7.48 | 6.29 | 6.29 | | 9.45 | 9.45 | 9.45 | 9.45 | 8.60 |
| Limestone | 5.57 | 5.57 | 5.97 | 6.79 | 6.79 | | 8.07 | 8.07 | 8.07 | 8.07 | 8.68 |
| Common salt | 4.47 | 4.47 | 3.92 | 2.82 | 2.82 | | 5.09 | 5.09 | 5.09 | 5.09 | 4.26 |
| Copper sulfate | 0.40 | 0.40 | 0.40 | 0.40 | 0.40 | | 0.40 | 0.40 | 0.40 | 0.40 | 0.40 |
| Zinc oxide | 2.50 | 2.50 | 2.50 | 2.50 | 2.50 | | 2.50 | 2.50 | 2.50 | 2.50 | 2.50 |
| L-Lysine – HCl 80% | 6.22 | 6.22 | 6.11 | 5.27 | 5.27 | | 5.26 | 5.26 | 5.26 | 5.26 | 4.79 |
| DL-Methionine 98% | 3.12 | 3.12 | 3.07 | 2.78 | 2.78 | | 2.60 | 2.60 | 2.60 | 2.60 | 2.43 |
| L-Threonine 98% | 3.23 | 3.23 | 3.13 | 2.66 | 2.66 | | 2.54 | 2.54 | 2.54 | 2.54 | 2.26 |
| L-Tryptophan 98% | 0.81 | 0.81 | 0.81 | 0.72 | 0.72 | | 0.54 | 0.54 | 0.54 | 0.54 | 0.50 |
| L-Valine 98% | 1.85 | 1.85 | 1.77 | 1.26 | 1.26 | | 1.06 | 1.06 | 1.06 | 1.06 | 0.76 |
| L-Isoleucine 99% | 0.60 | 0.60 | 0.74 | 0.69 | 0.69 | | 0.00 | 0.00 | 0.00 | 0.00 | 0.00 |
| Biocholine 60% | 0.20 | 0.20 | 0.20 | 0.20 | 0.20 | | 0.15 | 0.15 | 0.15 | 0.15 | 0.15 |
| Palatability enhancer | 0.30 | 0.30 | 0.30 | 0.30 | 0.30 | | 0.30 | 0.30 | 0.30 | 0.30 | 0.30 |
| Nutrients |  |  |  |  |  | |  |  |  |  |  |
| Metabolizable energy, kcal/kg | 3450 | 3450 | 3450 | 3450 | 3450 | | 3400 | 3400 | 3400 | 3400 | 3400 |
| Crude fat, % | 6.93 | 6.93 | 6.74 | 6.44 | 6.44 | | 6.33 | 6.33 | 6.33 | 6.33 | 6.09 |
| Crude fiber, % | 2.37 | 2.37 | 2.29 | 2.2 | 2.2 | | 2.7 | 2.7 | 2.7 | 2.7 | 2.61 |
| Lactose, % | 5.0 | 5.0 | 5.0 | 5.0 | 5.0 | | 0.0 | 0.0 | 0.0 | 0.0 | 0.0 |
| Crude protein, % | 19.71 | 19.71 | 19.66 | 20.2 | 20.2 | | 19.96 | 19.96 | 19.96 | 19.96 | 20.21 |
| Digestible Lysine, %^5^ | 1.4 | 1.4 | 1.4 | 1.4 | 1.4 | | 1.33 | 1.33 | 1.33 | 1.33 | 1.33 |
| Digestible Methionine, % | 0.56 | 0.56 | 0.55 | 0.52 | 0.52 | | 0.52 | 0.52 | 0.52 | 0.52 | 0.5 |
| Digestible Met + Cys, % | 0.84 | 0.84 | 0.84 | 0.84 | 0.84 | | 0.8 | 0.8 | 0.8 | 0.8 | 0.8 |
| Digestible Threonine, % | 0.94 | 0.94 | 0.94 | 0.94 | 0.94 | | 0.89 | 0.89 | 0.89 | 0.89 | 0.89 |
| Digestible Tryptophan, % | 0.28 | 0.28 | 0.28 | 0.28 | 0.28 | | 0.27 | 0.27 | 0.27 | 0.27 | 0.27 |
| Digestible Arginine, % | 1.15 | 1.15 | 1.13 | 1.14 | 1.14 | | 1.22 | 1.22 | 1.22 | 1.22 | 1.21 |
| Digestible Valine, % | 0.97 | 0.97 | 0.97 | 0.97 | 0.97 | | 0.91 | 0.91 | 0.91 | 0.91 | 0.91 |
| Digestible Isoleucine, % | 0.77 | 0.77 | 0.77 | 0.77 | 0.77 | | 0.74 | 0.74 | 0.74 | 0.74 | 0.74 |
| Digestible Leucine, % | 1.43 | 1.43 | 1.45 | 1.52 | 1.52 | | 1.51 | 1.51 | 1.51 | 1.51 | 1.56 |
| Digestible Histidine, % | 0,46 | 0,46 | 0,46 | 0,49 | 0,49 | | 0,47 | 0,47 | 0,47 | 0,47 | 0,49 |
| Digestible Phenylalanine, % | 0.8 | 0.8 | 0.8 | 0.84 | 0.84 | | 0.87 | 0.87 | 0.87 | 0.87 | 0.89 |
| Total Calcium, % | 0.75 | 0.75 | 0.75 | 0.75 | 0.75 | | 0.75 | 0.75 | 0.75 | 0.75 | 0.75 |
| Total Phosphorus, % | 0.65 | 0.65 | 0.65 | 0.65 | 0.65 | | 0.62 | 0.62 | 0.62 | 0.62 | 0.62 |
| Standardized available phosphorus, % | 0.45 | 0.45 | 0.45 | 0.45 | 0.45 | | 0.4 | 0.4 | 0.4 | 0.4 | 0.4 |
| Sodium, % | 0.25 | 0.25 | 0.25 | 0.25 | 0.25 | | 0.22 | 0.22 | 0.22 | 0.22 | 0.22 |
| Copper, mg/kg | 106 | 106 | 105 | 105 | 105 | | 107 | 107 | 107 | 107 | 107 |
| Zinc, mg/kg | 1849 | 1849 | 1848 | 1847 | 1847 | | 1487 | 1487 | 1487 | 1487 | 1486 |
| Choline, mg/kg | 302 | 302 | 311 | 327 | 327 | | 227 | 227 | 227 | 227 | 239 |

^1^ SDP, spray-dried plasma (AP920, APC do Brasil). ^2^Vitamin premix provided per kg of diet: 6,000 IU vitamin A; 1,500 IU vitamin D3; 15 mg vitamin E; 1.5 mg vitamin K3; 1.35 mg vitamin B1; 4 mg vitamin B2; 2 mg vitamin B6; 20 μg vitamin B12; 20 mg; 9.35 mg pantothenic acid; 600 μg folic acid; 80 μg biotin; 300 μg Se. ^3^Mineral premix provided per kg of diet: 100 mg Fe; 10 mg Cu; 40 g Mn; 1 mg Co; 100 mg Zn; 1.5 mg I. ^4^Product activity. ^5^Digestible amino acids values are expressed as standardized ileal digestible. CON = control group; L-SDP = low-inclusion spray-dried plasma; M-SDP = medium-inclusion spray-dried plasma; H-SDP = high-inclusion spray-dried plasma; VH-SDP = very-high-inclusion spray-dried plasma.

**Supplementary Table S3**. Ingredient and nutrient composition (as-fed matter basis) of the common diets for the growing and finishing phases.

| Ingredients, g/kg | Growing I | Growing II | Finisher I | Finisher II |
| --- | --- | --- | --- | --- |
| Corn, 7.88% CP | 693.3 | 715.3 | 740.9 | 754.4 |
| Soybean meal, 45% CP | 240.0 | 220.0 | 200.0 | 180.0 |
| Meat and bone meal, 41% CP | 25.0 | 25.0 | 25.0 | 20.0 |
| Soybean oil | 20.0 | 20.0 | 15.0 | 15.0 |
| Vitamin premix^1^ | 0.5 | 0.5 | 0.5 | 0.5 |
| Mineral premix^2^ | 1.0 | 1.0 | 1.0 | 1.0 |
| Phytase (10,000 FTU)^3^ | 0.05 | 0.05 | 0.05 | 0.05 |
| Limestone | 4.50 | 4.50 | 4.50 | 6.00 |
| Salt | 5.00 | 5.00 | 5.00 | 5.00 |
| Copper Sulfate | 0.40 | 0.30 | 0.30 | 0.30 |
| L-Lysine – HCl 80% | 4.79 | 4.10 | 4.05 | 4.10 |
| DL – Methionine 98% | 1.92 | 1.48 | 1.34 | 1.23 |
| L-Threonine 98% | 1.98 | 1.54 | 1.44 | 1.40 |
| L-Tryptophan 98% | 0.41 | 0.33 | 0.33 | 0.35 |
| L-Valine 98% | 0.68 | 0.29 | 0.26 | 0.29 |
| Choline chloride 60 | 0.40 | 0.40 | 0.25 | 0.25 |
| Enzyme carbohydrase | 0.10 | 0.10 | 0.10 | 0.10 |
| Nutrients |  |  |  |  |
| Metabolizable energy, kcal/kg | 3367 | 3367 | 3346 | 3351 |
| Crude fat, % | 5.24 | 5.29 | 4.85 | 4.84 |
| Crude Fiber | 2.47 | 2.40 | 2.34 | 2.28 |
| Crude protein, % | 18.1 | 17.2 | 16.5 | 15.6 |
| Digestible Lysine, %^4^ | 1.15 | 1.05 | 1.00 | 0.95 |
| Digestible Methionine, % | 0.43 | 0.38 | 0.36 | 0.34 |
| Digestible Met + Cys, % | 0.69 | 0.63 | 0.60 | 0.57 |
| Digestible Threonine, % | 0.77 | 0.70 | 0.67 | 0.64 |
| Digestible Tryptophan, % | 0.22 | 0.20 | 0.19 | 0.18 |
| Digestible Arginine, % | 1.07 | 1.01 | 0.96 | 0.89 |
| Digestible Valine, % | 0.79 | 0.72 | 0.69 | 0.66 |
| Digestible Isoleucine, % | 0.63 | 0.60 | 0.57 | 0.53 |
| Digestible Leucine, % | 1.40 | 1.36 | 1.32 | 1.27 |
| Digestible Histidine, % | 0.42 | 0.40 | 0.39 | 0.37 |
| Digestible Phenylalanine, % | 0.77 | 0.73 | 0.69 | 0.66 |
| Total Calcium, % | 0.74 | 0.73 | 0.73 | 0.71 |
| Total Phosphorus, % | 0.63 | 0.63 | 0.62 | 0.58 |
| Standardized available phosphorus, % | 0.40 | 0.40 | 0.40 | 0.36 |
| Sodium, % | 0.23 | 0.23 | 0.23 | 0.23 |
| Copper, mg/kg | 107 | 106 | 81 | 80 |
| Zinc, mg/kg | 28 | 27 | 27 | 26 |
| Choline, mg/kg | 240 | 240 | 150 | 150 |

^1^Vitamin premix provided per kg of diet: 6,000 IU vitamin A; 1,500 IU vitamin D3; 15 mg vitamin E; 1.5 mg vitamin K3; 1.35 mg vitamin B1; 4 mg vitamin B2; 2 mg vitamin B6; 20 μg vitamin B12; 20 mg; 9.35 mg pantothenic acid; 600 μg folic acid; 80 μg biotin; 300 μg Se. ^2^Mineral premix provided per kg of diet: 100 mg Fe; 10 mg Cu; 40 g Mn; 1 mg Co; 100 mg Zn; 1.5 mg I.
